# *Clostridium difficile* exposure as an insidious source of infection in healthcare settings: an epidemiological model

**DOI:** 10.1186/1471-2334-13-376

**Published:** 2013-08-16

**Authors:** Laith Yakob, Thomas V Riley, David L Paterson, Archie CA Clements

**Affiliations:** 1 School of Population Health, The University of Queensland, Brisbane, Australia; 2 School of Pathology and Laboratory Medicine, University of Western Australia, Crawley, Australia; 3 Centre for Clinical Research, The University of Queensland, Brisbane, Australia

## Abstract

**Background:**

*Clostridium difficile* is the leading cause of infectious diarrhea in hospitalized patients. Its epidemiology has shifted in recent years from almost exclusively infecting elderly patients in whom the gut microbiota has been disturbed by antimicrobials, to now also infecting individuals of all age groups with no recent antimicrobial use.

**Methods:**

A stochastic mathematical model was constructed to simulate the modern epidemiology of *C. difficile* in a healthcare setting, and, to compare the efficacies of interventions.

**Results:**

Both the rate of colonization and the incidence of symptomatic disease in hospital inpatients were insensitive to antimicrobial stewardship and to the prescription of probiotics to expedite healthy gut microbiota recovery, suggesting these to be ineffective interventions to limit transmission. Comparatively, improving hygiene and sanitation and reducing average length of stay more effectively reduced infection rates. Although the majority of new colonization events are a result of within-hospital ward exposure, simulations demonstrate the importance of imported cases with new admissions.

**Conclusions:**

By analyzing a wide range of screening sensitivities, we identify a previously ignored source of pathogen importation: although capturing all asymptomatic as well as symptomatic introductions, individuals who are exposed but not yet colonized will be missed by even a perfectly sensitive screen on admission. Empirical studies to measure the duration of this latent period of infection will be critical to assessing *C. difficile* control strategies. Moreover, identifying the extent to which the exposed category of individual contributes to pathogen importation should be explicitly considered for all infections relevant to healthcare settings.

## Background

*Clostridium difficile* is a Gram-positive, toxin-producing anaerobic bacterium. Worldwide, it is the leading cause of infectious diarrhea in hospitalized patients. The incidence and severity of *C. difficile* infection (CDI) varies considerably among studied populations but the general trend shows an increase in recent decades [1-3], with a higher proportion of CDI patients undergoing colectomy and dying [4,5]. The disease is currently estimated to cost $800 million per year in US acute care facilities [6]. Previously, persistently disturbed intestinal microbiota, usually as a result of antimicrobials, was considered a prerequisite of the disease. However, recent studies have demonstrated severe cases occurring in groups that were previously assumed to be low-risk, including pregnant women, children and people with no recent exposure to antimicrobials [7,8], indicating either a changing epidemiology or more testing in these groups [9].

There is increasing evidence for a potentially important role of asymptomatic carriage in the epidemiology of CDI [10], with increasing rates recorded within healthcare settings [11] and for the wider community [12,13]. Hospital rooms occupied by asymptomatic patients can have very high rates of contamination (29%) [14]. The dissociation between symptoms and infectivity was recently corroborated by a prospective clinical study in which 60% of patients still had skin contamination following resolution of diarrhea in CDI patients, and 37% continued to shed spores in their stool [15]. Carriers have been implicated in the global spread of hyper-virulent strains such as ribotype 027 which is believed to have caused over 2000 fatalities during the 2003–4 outbreak in Quebec, Canada [16]. Increased incidence, severity of disease associated with endemic strains and frequency of outbreaks emphasize the urgency for improved epidemiological understanding. To this end, we construct the most comprehensive epidemiological model of CDI transmission reported to date and use it to compare the efficacies of key interventions.

Surprisingly, there are only three modeling studies describing the mechanism of CDI transmission, each providing insight but with important limitations. The first mathematical model of CDI by Starr and colleagues [17] ignored asymptomatic carriage, a major potential source of infection for this disease [11,18]. While this omission was rectified by the authors in a subsequent study [19], differential rates of progression to symptomatic disease between patients who were and who were not taking antimicrobials [20] were not accounted for. The third modeling study [21], developed by Lanzas and colleagues, did not allow for symptomatic disease in patients who were not on antimicrobials thereby overlooking a growing body of research showing high incidence rates in previously healthy individuals to be a key feature of *C. difficile* epidemiology [22,23]. Although parsimony should always be a goal of epidemiological models, the inclusion of greater biological realism in our simulations highlights a hitherto unreported and potentially significant source of infection. We use this stochastic model to assess and compare the efficacies of several interventions to reduce infection transmission. We discuss the significance of our findings to infectious disease transmission within healthcare settings in general as well as specifically to the epidemiology of CDI.

## Methods

### The *C. difficile* pathogen

*C. difficile* is transmitted via the fecal-oral route. The infectious dose is small and the bacterium is capable of producing spores which can remain viable for years in the environment [24]. There has been a recent suggestion of inhalation of spores potentially providing a secondary mode of transmission [25,26], but this is unlikely to contribute significantly to the disease’s epidemiology. On ingestion, the pathogen colonizes the gut and the host typically sheds bacteria in their stool within two weeks.

A significant proportion of infections is asymptomatic, with a spectrum of symptoms of varying severity experienced by the remainder [14,27]. These symptoms include diarrhea, fever and abdominal pain and can result in toxic megacolon and death. Reasons for the development of symptomatic infection are incompletely understood but the risk factor most commonly associated with this progression is the disruption of normal gut microbiota by antimicrobials [28]. As a consequence of disrupted gut microbiota and lack of local immunity, the treatment of symptomatic infection with antimicrobials is met with high relapse rates in patients who do not successfully clear infection, as well as high reinfection rates [29]. Our model is the first that allows for treated individuals to return back to asymptomatically infected, exposed or uncolonized states to reflect the fact that treatment success is so variable. Following recent evidence of an increasing trend in colonization and disease in individuals who have not recently received antimicrobials [8], our model allows for the development of CDI without antibiotic involvement.

### Epidemiological model parameterization

**Figure 1:**
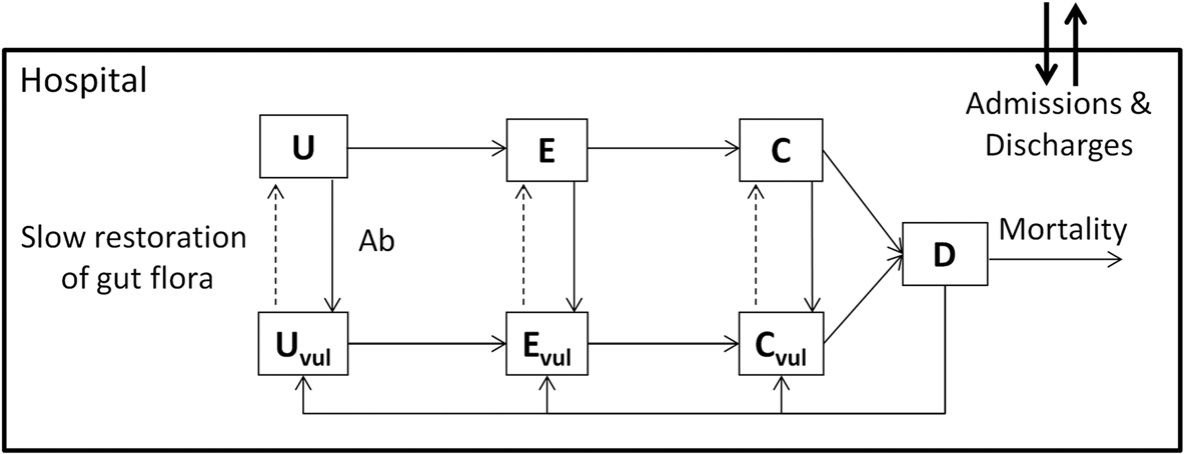
**Compartmental design of epidemiological model for *Clostridium difficile.*** Individuals are either ‘U’nexposed, ‘E’xposed, ‘C’olonized or ‘D’iseased and are increasingly ‘vul’nerable when they have taken antimicrobials (‘Ab’). Patients of all epidemiological states can be admitted but only discharged if not symptomatic.

Patients in a hospital with 1000 beds were simulated for 1 year. Individuals were stratified according to infection status (Unexposed, Exposed, Colonized or Diseased) and whether or not they were currently taking antimicrobials (or had taken antimicrobials within the last 3 months). Exposed, colonized and diseased patients all harbor the pathogen; colonized and diseased patients contribute to transmission, but only diseased patients exhibit symptoms. The overall structure of the model is described in Figure 1. One novel component of our study is the incorporation of an ‘Exposed’ class of individuals which explicitly allows for the fact that individuals who are not colonized on admission may subsequently become colonized regardless of within-hospital exposure. The epidemiological consequences of this addition are discussed fully after the analysis.

Average length of stay was assumed to be 6 days, reflecting rates that are typical of the US, Europe and Australia [30]. Patients could be admitted in any of the seven possible states but only discharged if they were not suffering from symptomatic disease. The proportion of new admissions that was taking (or had recently taken) antimicrobials was assumed to be 25% and the rates of antimicrobial prescription assumed that 50% of the hospital inpatients were currently on antimicrobials (or had taken them in the previous 3 months) [31,32]. Recovery of gut microbiota following cessation of antimicrobial treatment was assumed to take 3 months [33]. New admissions were assumed to perfectly balance discharges, resulting in a constant population size that experienced frequency-dependent transmission. This is typical for the epidemiological modeling of human infectious diseases [34].

After a period of 5 days, exposed individuals became asymptomatically colonized [35]. For patients on antimicrobials, progression to symptomatic disease took a further 5 days [20]. Patients not on antimicrobials were assumed to be five-times less likely to progress to symptomatic disease [20]. Cure rate following treatment, which took 10 days [36], was assumed to be 80% [37], with the remaining treated patients returning to the exposed category. The symptoms of 33% of patients with CDI self-resolved [38] and 2% of symptomatic CDI patients died [20]. Table 1 summarizes the model parameters and definitions with associated cited studies.

**Table 1:** Epidemiological model symbology and parameterization

**Parameter**	**Definition**	**Value (,Vul)**	**Reference**
***λ***	Recovery of gut microbiota (day^-1^)	0.011	33
***α***	Antibiotic treatment (day^-1^)	0.11	31,32
***β***	Exposure (day^-1^)	Full range SIM	
***η***	Dev into asympt infectious (day^-1^)	0.2,0.2	35
***θ***	Dev symptomatic CDI (day^-1^)	0.04,0.2	20
***ρ***	CDI treatment (day^-1^)	0.1	36
***σ***	Treatment failure (prop.)	0.2	37
***ζ***	Self-resolving symptoms (prop.)	0.33	38
***μ***	CDI case fatality rate (prop.)	0.02	20
***κ***	Hospital discharge (day^-1^)	0.17	30
***ϵ***	Hospital admission (proportion)	0.75,0.25	31,32
***Q***	Quarantined CDI ‘0’ yes, ‘1’ no	n/a	

### Mathematical model construction

Ordinary differential equations were constructed to reflect the biology of infection as parsimoniously as possible (Additional file 1). Traditionally, the basic reproduction number, R0, is used to estimate transmission potential of an infectious disease. R0 is a useful metric for estimating outbreak thresholds and is therefore of relevance in estimating risk of epidemic strains introduced into fully susceptible (disease-free) populations. The stable endemicity of *C. difficile* within healthcare settings represents a significant departure from a disease-free population. Moreover, R0 no longer quantifies risk nor discriminates between high levels and low levels of transmission when constant imports of pathogen are accounted for in healthcare settings [39]. Therefore, we present our results of control simulations in terms of the incidence of symptomatic disease and the ratio of colonized patients discharged from hospital relative to patients colonized on admission. An advantage of these measures of intervention efficacy is that they can be directly measured.

Because the simulated population is small, infection dynamics are more likely to be governed by stochastic processes. Therefore, the ordinary differential equations were converted into a stochastic simulation system based on Gillespie’s direct method [40]. This method of simulation accounts for continuous time but discrete state space whereby the probability of conversion from one state (e.g., Exposed) to another (e.g., Colonized) is determined by the associate rate of the ordinary differential equation system. There are 15 possible epidemiological state transitions in this system and they are summarized in Table 2. The table shows the transfer of individuals from one epidemiological compartment (“-1”) to another (“+1”).

**Table 2:** Epidemiological state transitions of the event-driven, stochastic model

**Event**	**Transition probability**	**State change**
**Antibiotic treatment of** uncolonized individual	*αUδt*	*U*-1, *U*_*v*_+1
exposed individual	*αEδt*	*E*-1, *E*_*v*_+1
colonized individual	*αCδt*	*C*-1, *C*_*v*_+1
**Gut microbiota recovery of** uncolonized individual	*λU*_*v*_*δt*	*U*_*v*_-1, *U*+1
exposed individual	*λE*_*v*_*δt*	*E*_*v*_-1, *E*+1
colonized individual	*λC*_*v*_*δt*	*C*_*v*_-1, *C*+1
**Exposure of** individual without recent Ab use	*β*[(*C+C*_*v*_*+DQ*)*U/N*]*δt*	*U*-1, *E*+1
individual with recent Ab use	*β*[(*C+C*_*v*_*+DQ*)*U*_*v*_*/N*]*δt*	*U*_*v*_-1, *E*_*v*_+1
**Colonization of** individual without recent Ab use	*ηEδt*	*E*-1, *C*+1
individual with recent Ab use	*ηE*_*v*_*δt*	*E*_*v*_-1, *C*_*v*_+1
**Symptoms develop in** indiv. without recent Ab use	*θCδt*	*C*-1, *D*+1
individual with recent Ab use	*θ*_*v*_*Cvδt*	*C*_*v*_-1, *D*+1
**Symptoms resolve**	*ζDδt*	*D*-1, *C*_*v*_+1
**Full clearance following treatment**	(1-*σ*)*ρD*δt	*D*-1, *U*_*v*_+1
**Treatment does not result in full clearance**	*σρD*δt	*D*-1, *E*_*v*_+1

This stochastic model was used to compare the efficacy of four different control measures in reducing the rate of colonization within the hospital as well as the incidence of symptomatic CDI. These controls were 1) reducing the rate of antimicrobial prescription in order to minimize the proportion of patients with heightened vulnerability to symptomatic disease, 2) reducing the level of environmental contamination and improving hand hygiene, 3) administering probiotics to expedite the restoration of healthy gut microbiota, and, 4) the reduction of average length of stay of inpatients.

The importance of asymptomatic patients in infection transmission is a topic that has received a lot of recent interest [10,11,41,42]. Despite this, the incubation periods for *C. difficile* are less well defined than for other pathogens [9]. Simulating infection dynamics across a wide range of incubation periods, we assessed the applicability of screening for asymptomatic, as well as symptomatic, admissions. Recent developments in commercially-available PCR screening technology have provided a sensitive (~90%) tool for rapidly detecting (~2 h) colonization with *C. difficile*[43]. Therefore, we gauged the utility of this screen as a further method of intervention under the assumption that hospitals have the facilities to segregate colonized (both asymptomatic and symptomatic) and non-colonized patients.

## Results and discussion

**Figure 2:**
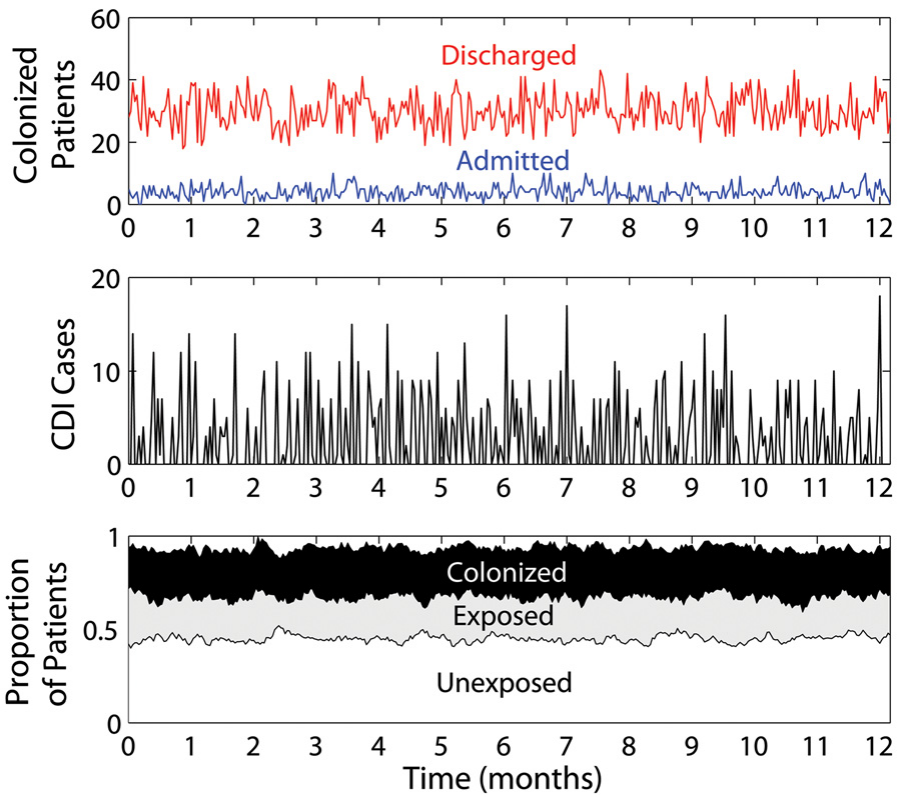
**A typical simulation using baseline model parameterization.** Subplots show the percentage of *Clostridium difficile* colonized patients who are admitted and discharged (top), the incidence of symptomatic disease (middle) and the proportional distribution of inpatient infection status (bottom). Stochastic (Direct Gillespie algorithm) simulations are run for a hospital with 1000 beds whereby newly admitted patients perfectly balance discharged individuals.

The temporal output from a typical stochastic simulation run, using baseline parameterization and in the absence of intervention is shown in Figure 2. The top panel shows the percentage of colonized individuals discharged compared with the percentage of colonized admissions. Average colonization prevalence on admission was 4.0% (st. dev. 2.0) which closely matches a recent Canadian study that measured asymptomatic admission prevalence of 4.4% in 6 hospitals in Quebec 2006–2007 [20]. We could not find clinical studies measuring the prevalence of colonization on discharge to directly corroborate our simulated values. However, in our study, colonization and length of stay are independent variables and so prevalence in discharged patients is representative of inpatient prevalence. Hospital inpatient prevalence of endemic *C. difficile* varies a great deal over time and between different locations, but our average colonization prevalence of 30% (st. dev. 5.6%) falls within the range described in the clinical literature [44].

The middle panel of Figure 2 shows the incidence of symptomatic *C. difficile* infection which averages 2.8 cases per 1000 hospital bed days (st. dev. 4.2), also within the range described in the most comprehensive pan-European survey [45]. The very high rates of underreporting that are known to occur for this pathogen, especially in younger patients who have less severe symptoms, suggest that the true prevalence of infection lies towards the top-end of estimates [46]. The bottom panel shows the proportional distribution of the epidemiological states of hospital inpatients in which the ratio of total (vulnerable or otherwise) Unexposed: Exposed: Colonized: Diseased patients is 0.45: 0.26: 0.24: 0.05. Importantly, our baseline parameterization using the most up-to-date data available for this pathogen suggests that there are as many individuals in the ‘Exposed’ category as in the ‘Colonized’ category.

**Figure 3:**
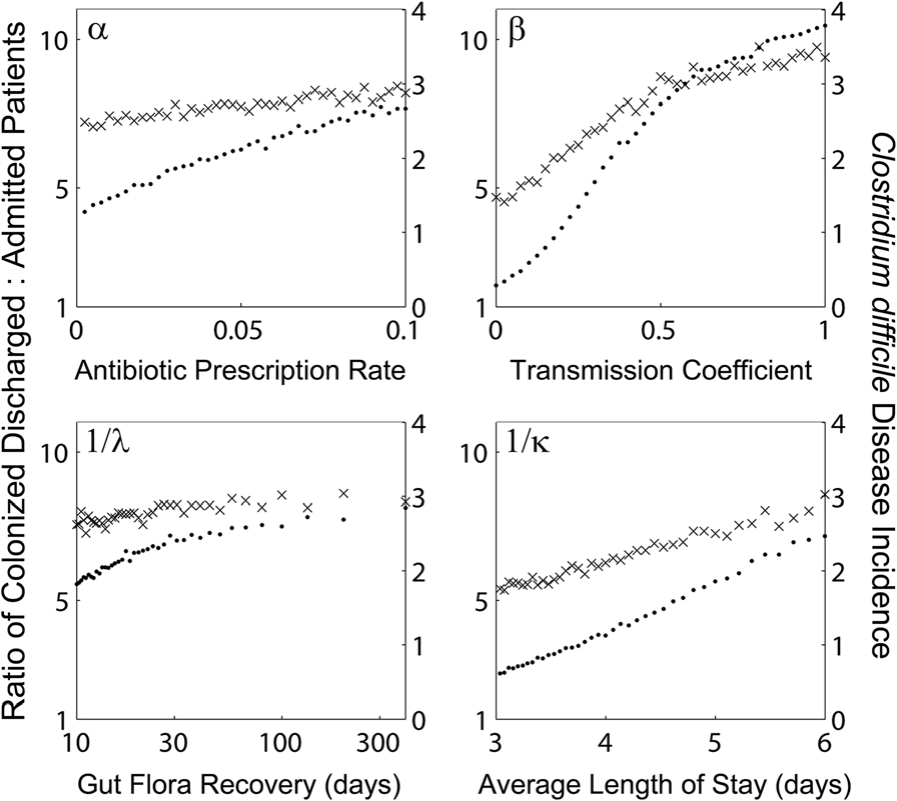
**The effects of different control measures on *Clostridium difficile *in the simulated hospital.** The ratio of patients colonized with *C. difficile* when discharged compared to new admissions (left Y axis, points) are shown along with associated incidence of symptomatic disease per 1000 hospital bed days (right Y axis, crosses) across a wide range of the four tested control measures: reducing antimicrobial prescription (top-left), improved sanitation and hygiene (top-right), administering pro-biotics (bottom-left) and reducing the average length of stay (bottom-right). Each point is the average value of a 1-year simulation.

Figure 3 shows the sensitivity of two different outputs (ratio of colonized patients discharged compared with on admission, and, the incidence of symptomatic CDI), to the four model parameters pertaining to control – namely, the rate of antimicrobial prescription, the transmission coefficient (reduced by improved hygiene and sanitation), gut microbiota recovery rate (expedited with probiotics) and average length of stay. Projections from the model reinforce the clinical evidence supporting a reduced length of stay [47,48] and infection control [49-51] as effective methods for attenuating the spread of *C. difficile*. While there is reassuring agreement between our model projections and clinical records of intervention efficacy, there is also an important disparity worth noting. Several recent studies have suggested antimicrobial stewardship to be an effective method of reducing the rate of CDI in hospitals [52-55]. However, these include studies that examined stewardship in conjunction with infection control procedures [54,55], thereby obscuring the efficacy of reduced prescription rates alone. They also include studies that found either borderline significant reduction in CDI (P=0.04, [53]) or no statistical significance through a reduction in either high-risk drugs or total antimicrobial usage in healthcare settings (respectively, P=0.0597 and P=0.0823, [52]). Therefore, the conflicting evidence for CDI reduction through antimicrobial stewardship is in keeping with a small effect size, as demonstrated by our simulations.

Infection rates were also insensitive to increased gut microbiota recovery promoted by probiotics. Cautious interpretation of this result is urged because while we found the overall influence that this treatment has on population-level transmission dynamics to be minimal, it does not necessarily negate the individual-level health benefits of probiotics.

Comparatively much greater returns were achieved by reducing the length of stay and through improved hygiene (respectively, bottom-right and top-right panel Figure 3). Halving the average inpatient length of stay yielded almost a 3-fold reduction in the colonized ratio of discharged compared with admitted patients. Additionally, a 10-fold decrease in CDI incidence was theoretically possible through improved hygiene and sanitation practices. However, even in this scenario whereby complete elimination of within-ward exposure was achieved (β=0), more patients left the hospital colonized than when they arrived. This is due to the expedited progression of disease resulting from antimicrobials, suggesting that stewardship might come into play as a more influential control tool once initial efforts have already eliminated ward-based transmission.

A thorough sensitivity analysis of model input parameters (see Additional file 2) demonstrates the robustness of our results pertaining to infection control. However, there are several limitations of this study that will require additional development and refinement. The life-course of the pathogen is incompletely understood. Our model structure assumes that all individuals who become symptomatic must first pass through a phase of shedding the pathogen asymptomatically. This follows on from the very comprehensive epidemiological study carried out by Loo and coauthors in which the time to developing symptomatic disease was measured to be twice that for developing asymptomatic colonization [20]. An alternative interpretation of this finding would be that the individuals who develop symptomatic infection never pass through a phase of asymptomatic shedding, but, instead, have a delayed progression of colonization. However, this would imply that successful colonization within patients that develop symptomatic disease is somehow delayed. In the absence of definitive evidence, therefore, our assumption seems more likely. Another limitation of the study is the assumption that asymptomatic shedders contribute equivalently to transmission as symptomatically infected individuals. While symptomatic disease involves a greater bacterial load, asymptomatic shedding would not prompt the same level of cautiousness in hygiene practices. Ascertaining the relative contributions of both category of individual will be a difficult, but important, issue to reconcile. Once good, reproducible data become available on this difference, the model can easily be adjusted to incorporate the additional information. A further limitation is the lack of consideration for healthcare professionals (and, also, hospital visitors) as vectors of infection – again, something that will require further development when the epidemiological data become available. Greater complexity can be iteratively incorporated into the framework presented here to explore additional risk factors including patient age as well as underlying co-morbidities and patient immune status. Future directions for this work also include assessing the integration of control tools. Integrated control has been strategized with mathematical models for a number of infectious diseases including sexually transmitted infections [56,57], vector-borne diseases [58-60], newly emerging infections and pathogenic bioterrorism attacks [61,62]. However, research into strategic combinations of healthcare-acquired infection control methods is underdeveloped.

**Figure 4:**
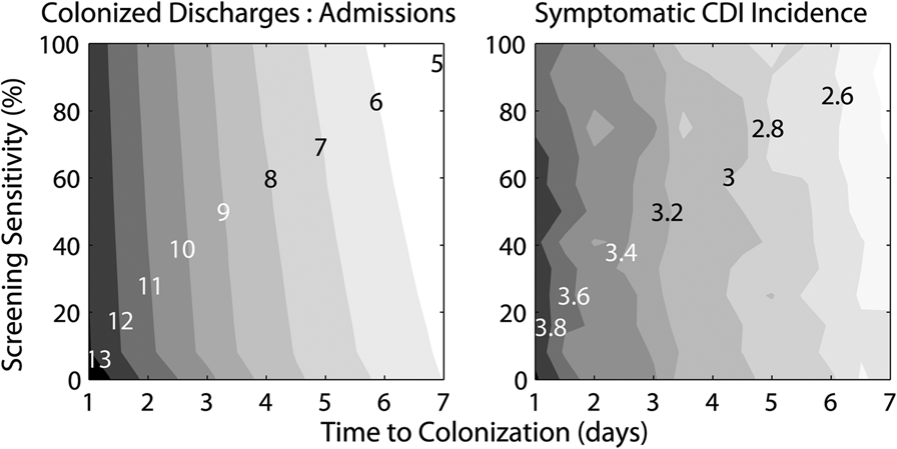
**The effects of screening on *Clostridium difficile *in the simulated hospital.** The ratio of patients that are colonized with *C. difficile* when discharged relative to on admission (left) are shown along with the associated incidence in symptomatic CDI per 1000 hospital bed days (right). Even highly sensitive screening (Y axis) can do little to impact either of these epidemiological outcomes. However, time to colonization (X axis), a component of the pathogen’s life course for which data is absent, is highly influential in its epidemiology.

Finally, we measured the sensitivity of the colonization ratio and the incidence of CDI on the pathogen’s incubation period – the *C. difficile* life history parameter of greatest uncertainty [9]. Intuitively, for shorter incubation periods, the colonization ratio increased along with CDI incidence (Figure 4). This is because longer incubation periods delay the rate at which a pathogen can spread between individuals. Remarkably, however, infection rates were extremely insensitive to screening sensitivity. Even where screening was 100% sensitive (current leading rapid screen sensitivity is ~90%) [43], little impact on infection rates was achieved by the segregation of all colonized admissions from non-colonized inpatients. Recent studies have suggested that a large proportion of patients colonized with *C. difficile* in healthcare settings was likely exposed to the pathogen before they were admitted [11,21,41,42]. These authors thereby attribute a substantial role of asymptomatic individuals on the importation of infection. In this analysis, by screening and subsequently isolating both asymptomatic and symptomatic patients from the non-colonized inpatients, we have eliminated these sources of infection and uncovered a previously ignored and potentially important source of *C. difficile* in healthcare settings: new admissions who have been exposed to the pathogen but not yet colonized.

## Conclusions

Even a perfect screening test giving immediate results for all new admissions will fail to detect the carriage of *C. difficile* into the hospital by patients. Although obtaining good estimates of the incubation period has proven particularly difficult [9], it will be critical to uncovering the extent to which the exposed category of individuals influences the epidemiology of CDI. This finding is not only relevant to *C. difficile* epidemiology, but to any pathogen that can be undetectably transported by exposed individuals.

## Competing interests

The authors declare that they have no competing interests.

## Authors’ contributions

LY, TVR, DLP and ACAC contributed towards the design of the model. LY constructed and analyzed the model. LY, TVR, DLP and ACAC wrote initial drafts of the manuscript. LY, TVR, DLP and ACAC read and approved the final manuscript.

## Pre-publication history

The pre-publication history for this paper can be accessed here:

http://www.biomedcentral.com/1471-2334/13/376/prepub

## Supplementary Material

Additional file 1 **Ordinary differential equations describing the framework of *Clostridium difficile *infection transmission.** Click here for file

Additional file 2 **Sensitivity analysis of the stochastic *Clostridium difficile *mathematical model.** Click here for file
