# Supplementary material for: Clostridium difficile exposure as an insidious source of infection in healthcare settings: an epidemiological model
Source: BMC Infect Dis. 2013 Aug 16;13:376. doi: 10.1186/1471-2334-13-376 (PMC3751620; doi:10.1186/1471-2334-13-376)
Supplement: Additional file 1 — Ordinary differential equations describing the framework of Clostridium difficile infection transmission. [file 1471-2334-13-376-S1.docx]

**Supplementary Material**

The ordinary differential equations describing the instantaneous rates of change between the seven possible epidemiological states (where the total hospital inpatient population, N=U+Uv+E+Ev+C+Cv+D):

$$\frac{dU}{dt}=\varepsilon_{U}\kappa N+\lambda U_{v}-\beta\frac{\left( C+C_{v}+DQ \right)U}{N}-\left( \alpha+\kappa\right)U$$

$$\frac{dU_{v}}{dt}=\varepsilon_{Uv}\kappa N+\alpha U+(1-\sigma)\rho D-\beta\frac{\left( C+C_{v}+DQ \right)U_{v}}{N}-\left( \lambda+\kappa\right)U_{v}$$

$$\frac{dE}{dt}=\varepsilon_{E}\kappa N+\lambda E_{v}+\beta\frac{\left( C+C_{v}+DQ \right)U}{N}-\left( \alpha+\eta+\kappa\right)E$$

$$\frac{dE_{v}}{dt}=\varepsilon_{Ev}\kappa N+\alpha E+\beta\frac{\left( C+C_{v}+DQ \right)U_{v}}{N}+\sigma\rho D-\left( \lambda+\eta+\kappa\right)E_{v}$$

$$\frac{dC}{dt}=\varepsilon_{C}\kappa N+\lambda C_{v}+\eta E-\left( \alpha+\theta+\kappa\right)C$$

$$\frac{dC_{v}}{dt}=\varepsilon_{Cv}\kappa N+\alpha C+\eta E_{v}+\zeta D-\left( \lambda+\theta_{v}+\kappa\right)C_{v}$$

$$\frac{dD}{dt}=\varepsilon_{D}\kappa N+\theta C+\theta_{v}C_{v}-\left( \zeta+\rho+\mu\right)D$$
