# Supplementary material for: Clostridium difficile exposure as an insidious source of infection in healthcare settings: an epidemiological model
Source: BMC Infect Dis. 2013 Aug 16;13:376. doi: 10.1186/1471-2334-13-376 (PMC3751620; doi:10.1186/1471-2334-13-376)
Supplement: Additional file 2 — Sensitivity analysis of the stochastic Clostridium difficile mathematical model. [file 1471-2334-13-376-S2.docx]

Sensitivity Analysis of *Clostridium difficile* Mathematical Model

Sensitivity was analysed for the incubation period (η), the rate of symptoms onset (θ), the CDI treatment failure rate (σ), the proportion of admissions in the vulnerable categories (ξ_v_) and the rate that symptoms self-resolve (ζ), across mean value minus 10% (‘Low’), mean value (‘Mid’) and mean value plus 10% (‘High’). The plots show the means (points) and standard deviations (error bars) of the rates of *C. difficile* disease incidence (CDI) and the ratio of patients colonized when discharged relative to when admitted (Colonization Ratio) calculated for simulation runs of 1000 days.


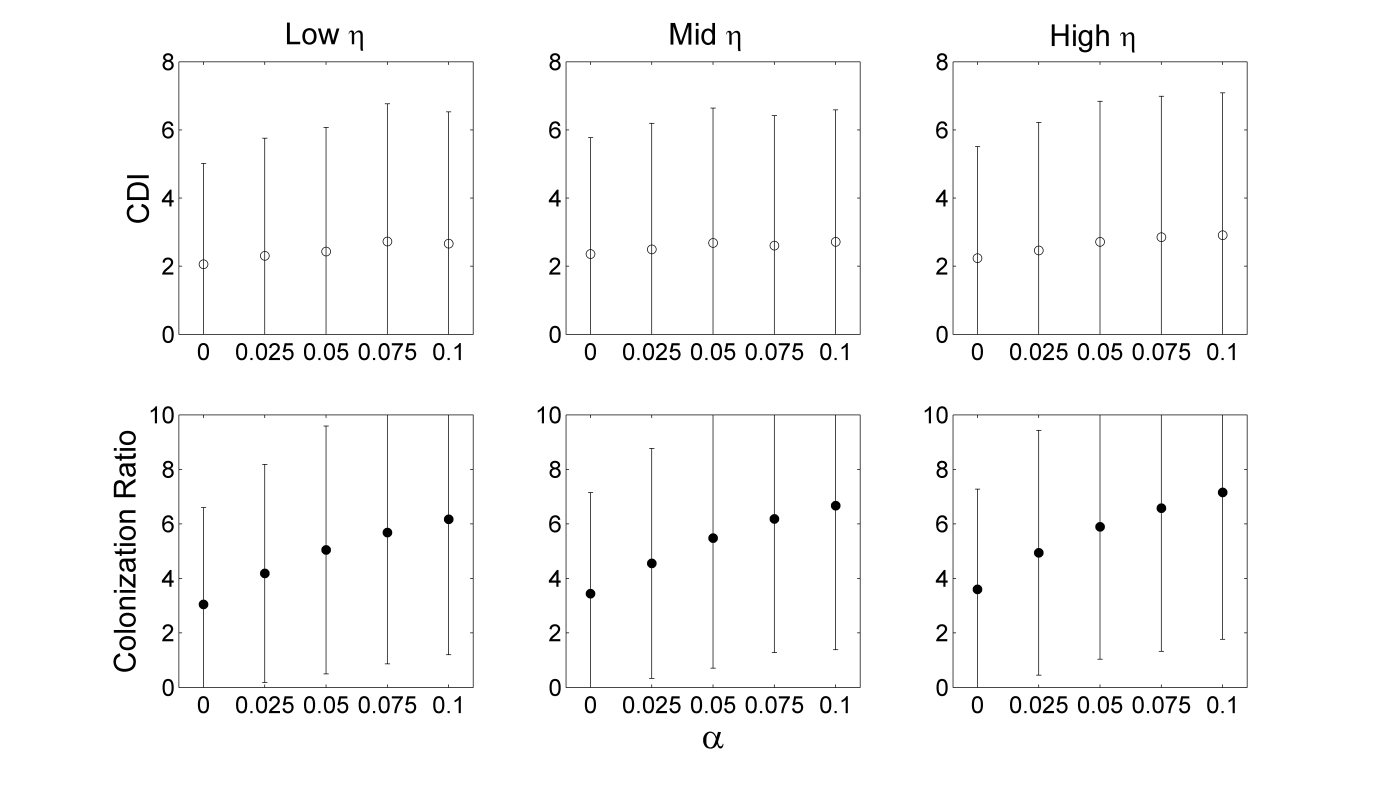


Supplementary Figure 1. Antimicrobial stewardship (α) sensitivity to the incubation period (η).


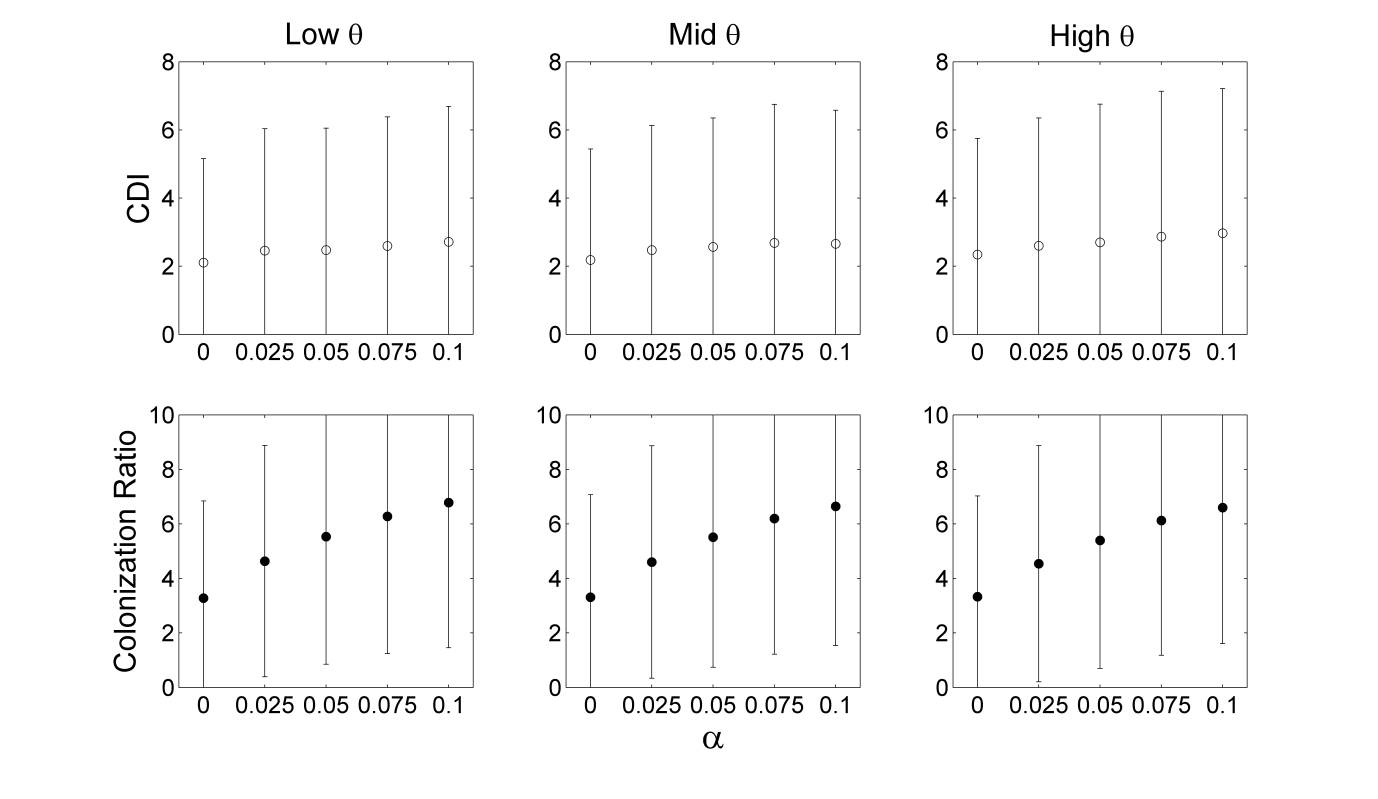
 Suppl Fig 2. Antimicrobial stewardship (α) sensitivity to the rate of symptoms onset (θ).


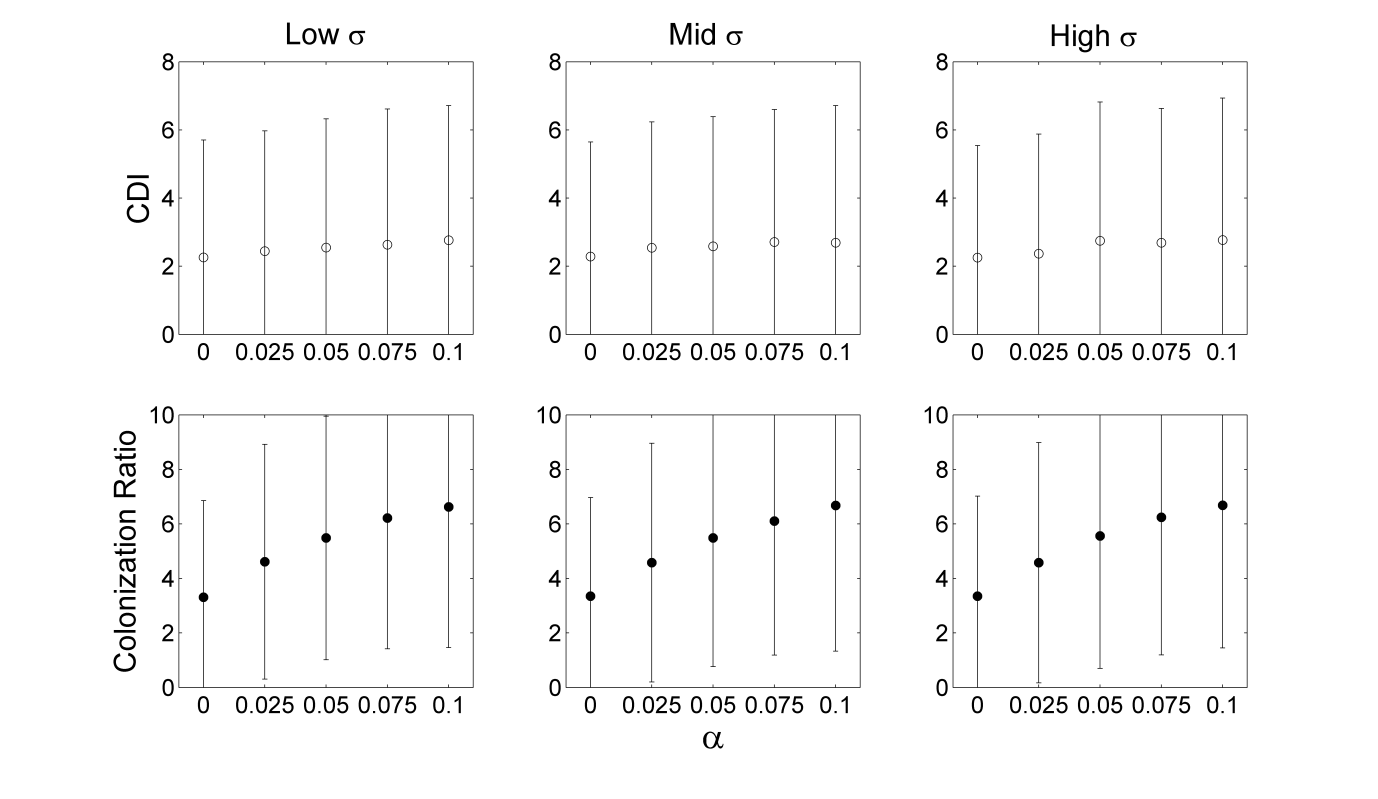


Suppl Fig 3. Antimicrobial stewardship (α) sensitivity to the rate of CDI treatment failure (σ).


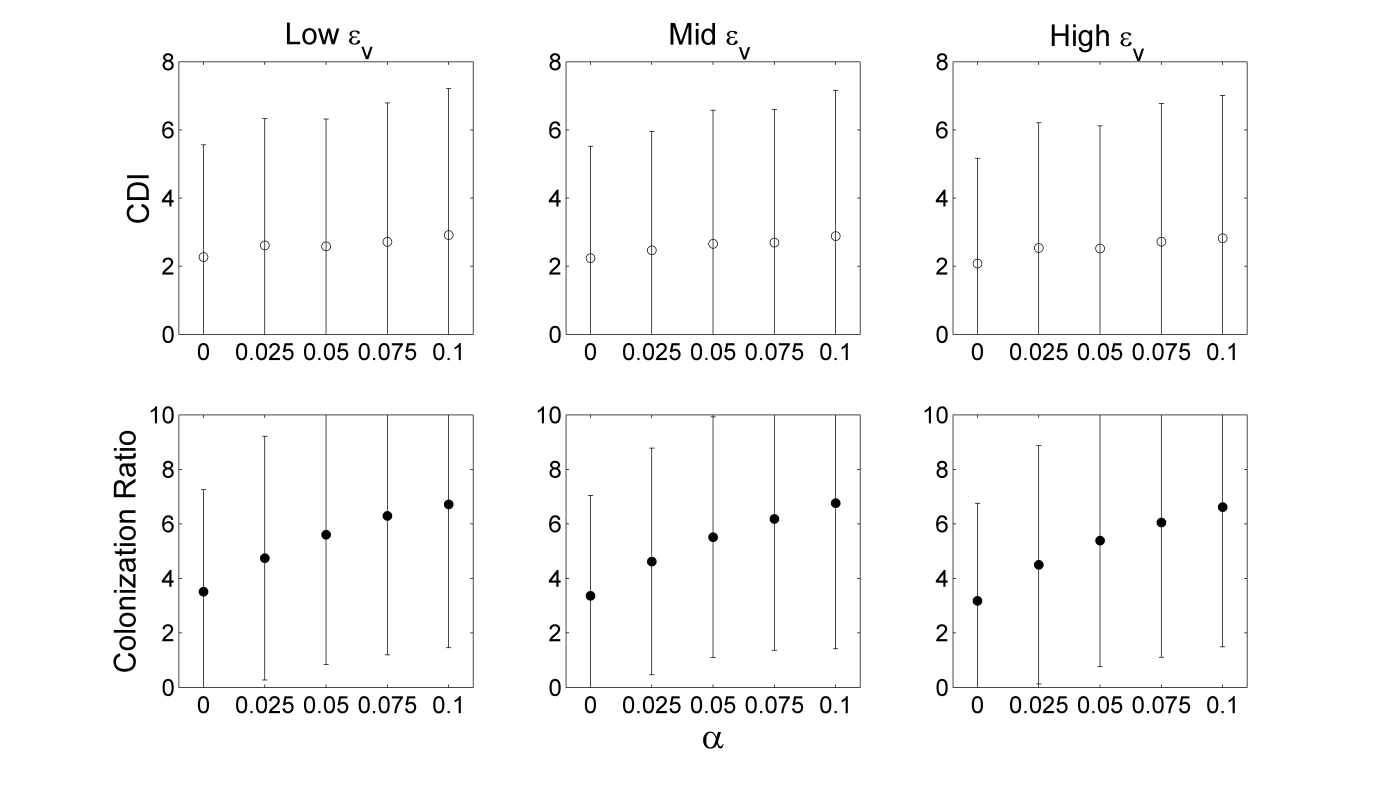


Suppl Fig 4. Antimicrobial stewardship (α) sensitivity to the proportion of vulnerable admissions (ε_v_).


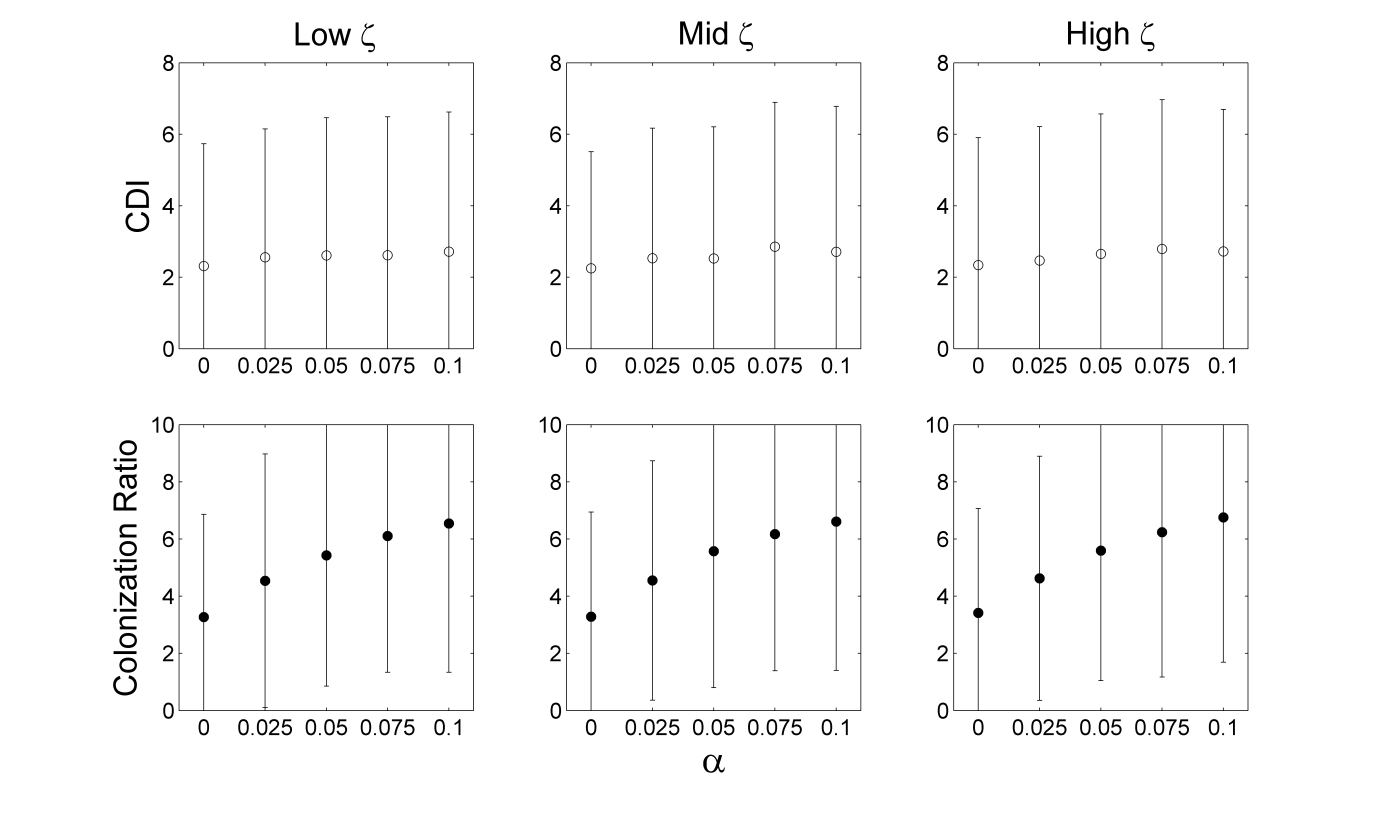


Suppl Fig 5. Antimicrobial stewardship (α) sensitivity to the rate of self-resolved symptoms (ζ).


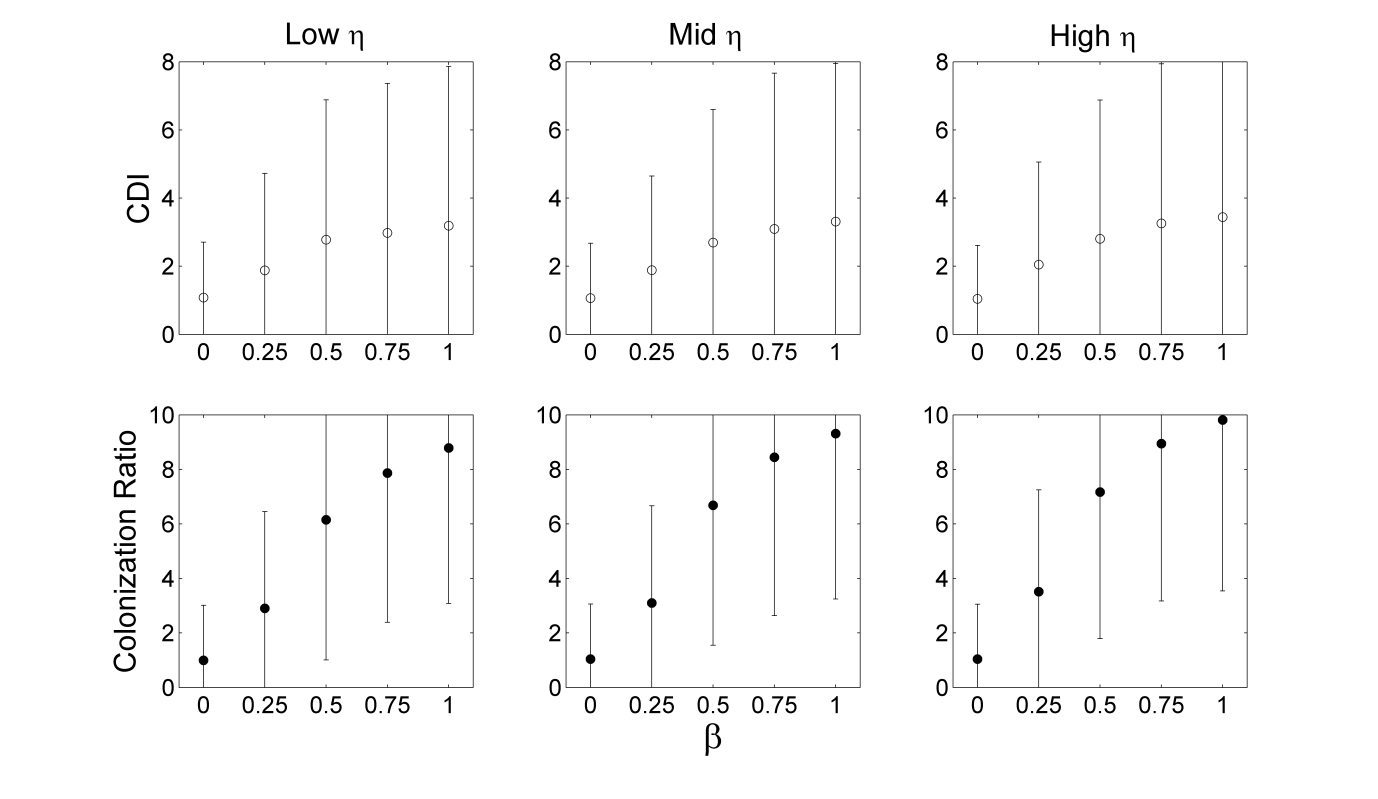


Suppl Fig 6. Improved hygiene and sanitation (β) sensitivity to the incubation period (η).


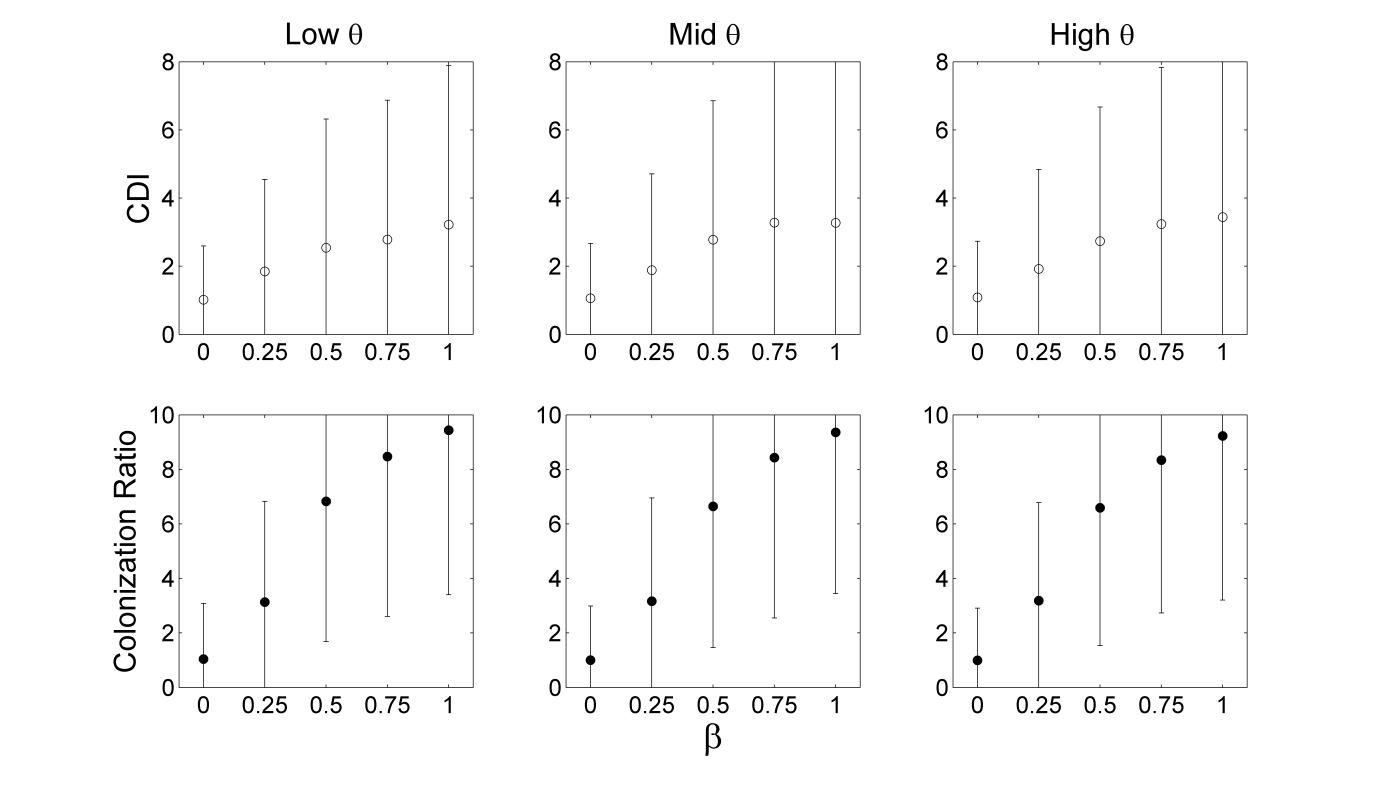


Suppl Fig 7. Improved hygiene and sanitation (β) sensitivity to the rate of symptoms onset (θ).


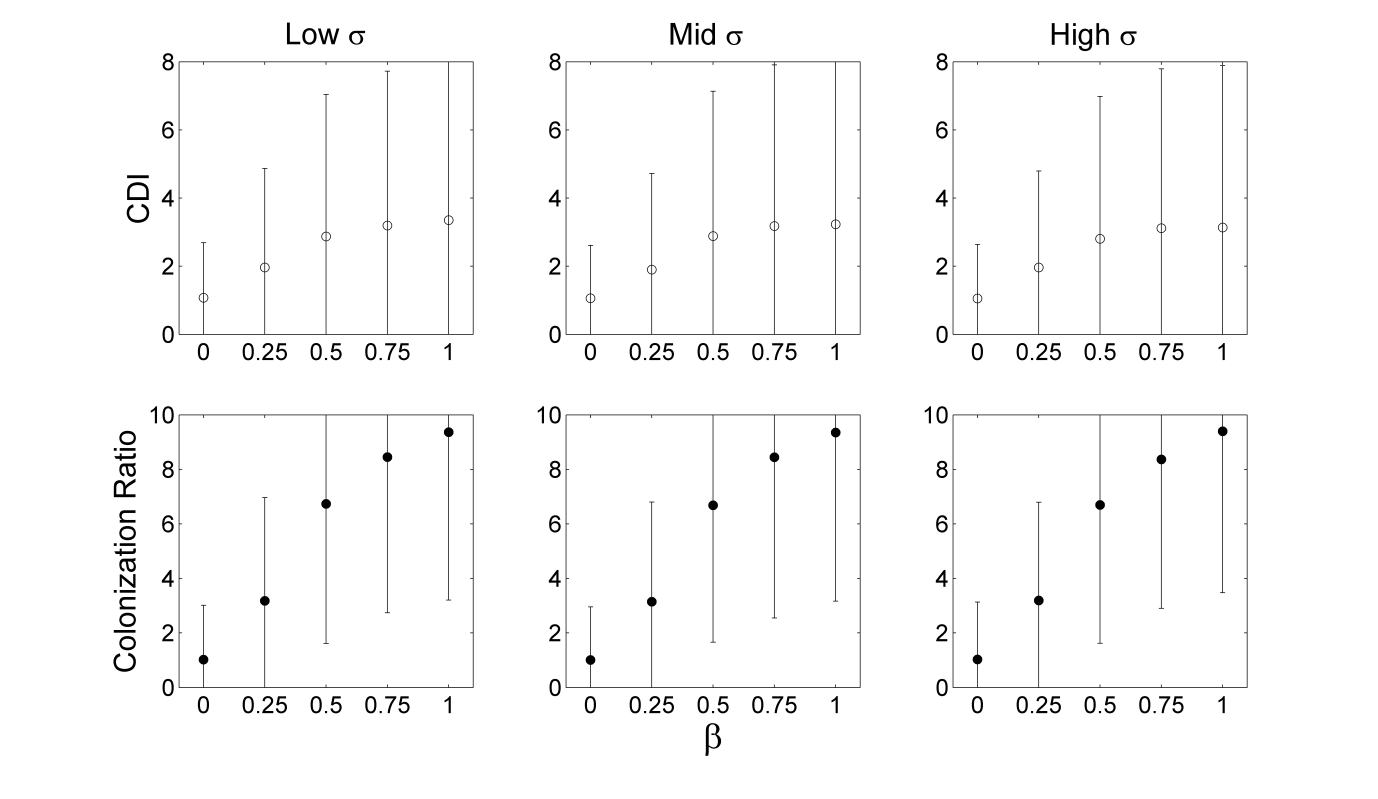


Suppl Fig 8. Improved hygiene and sanitation (β) sensitivity to the CDI treatment failure rate (σ).


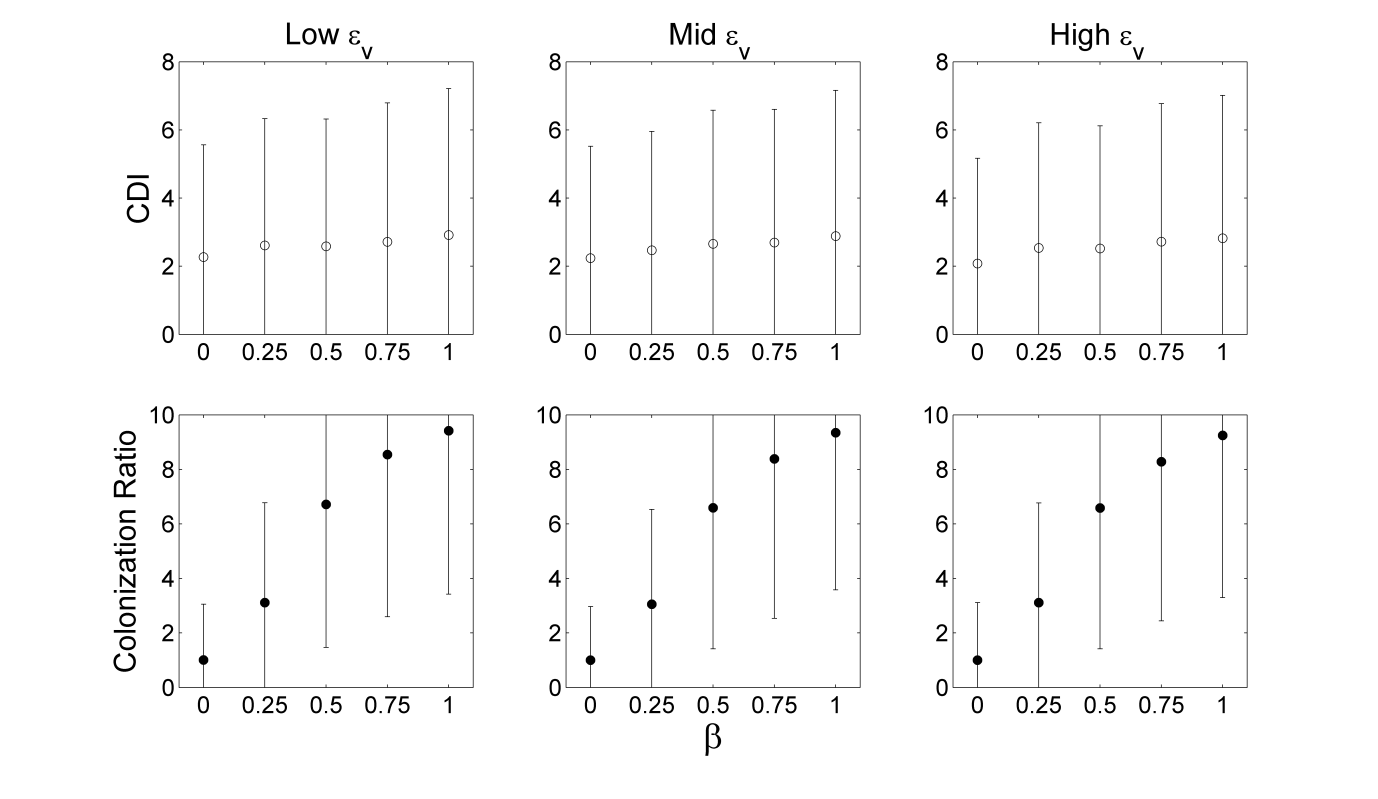


Suppl Fig 9. Improved hygiene and sanitation (β) sensitivity to the proportion of vulnerable admissions (ε­_v_).


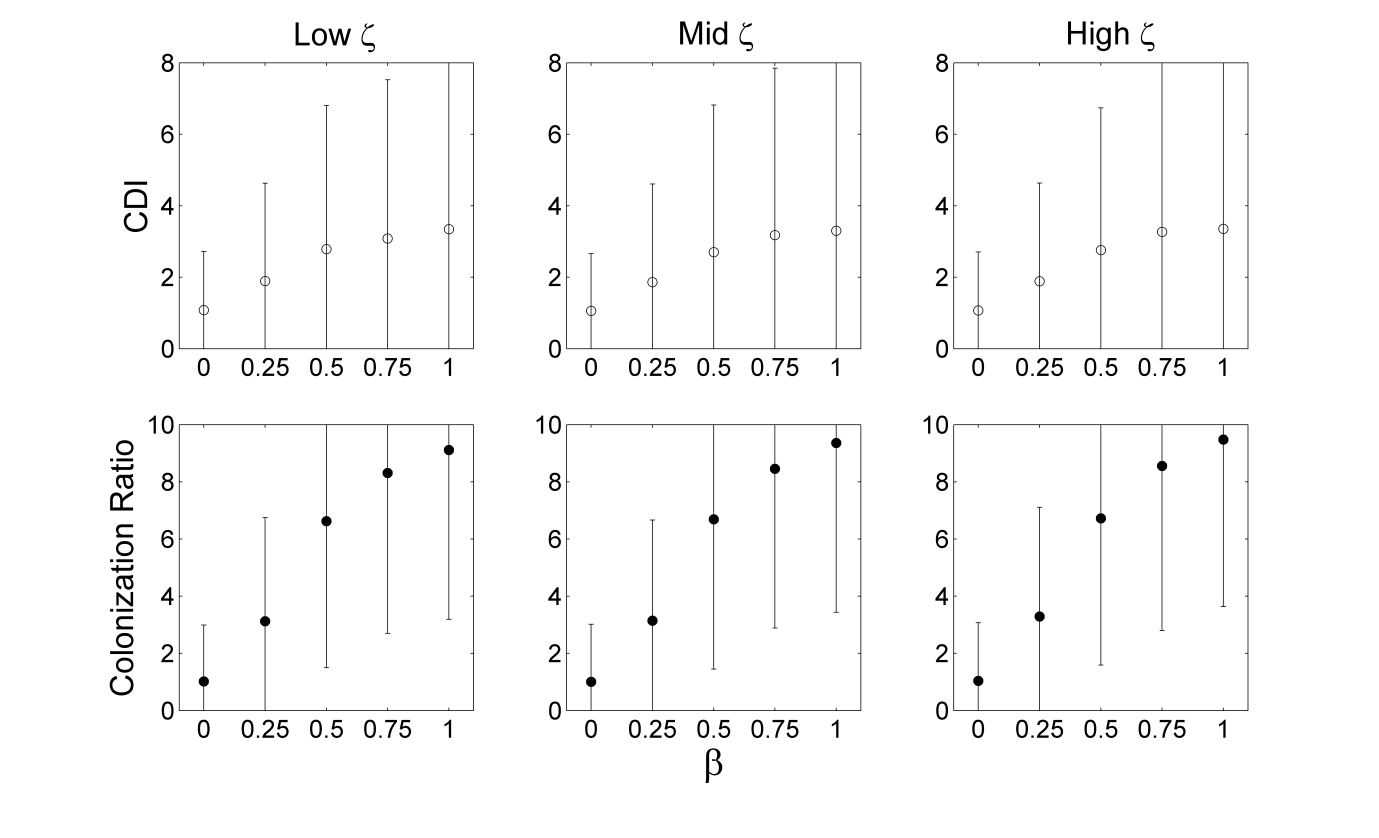


Suppl Fig 10. Improved hygiene and sanitation (β) sensitivity to rate of self-resolved symptoms (ζ).


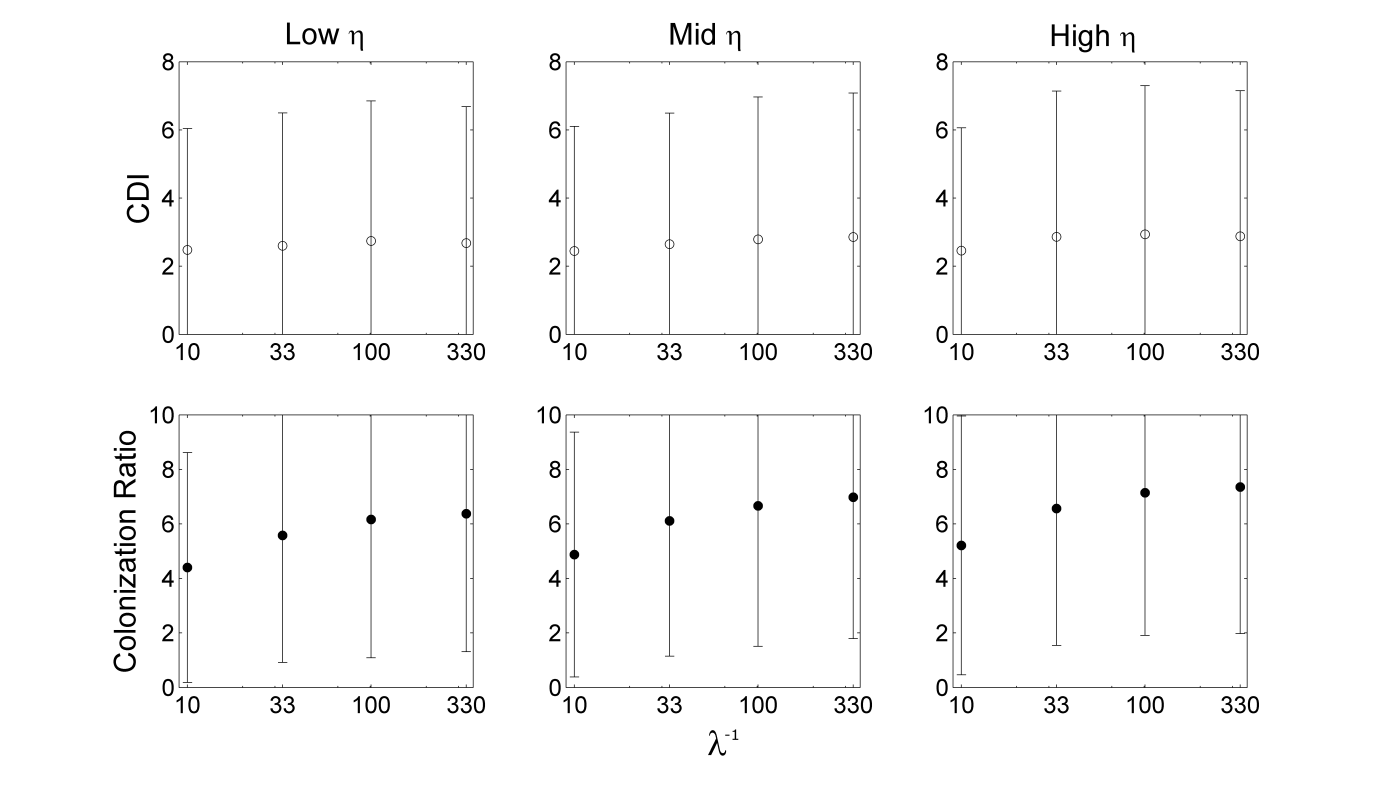


Suppl Fig 11. Probiotic gut restoration (λ^-1^) sensitivity to the incubation period (η).


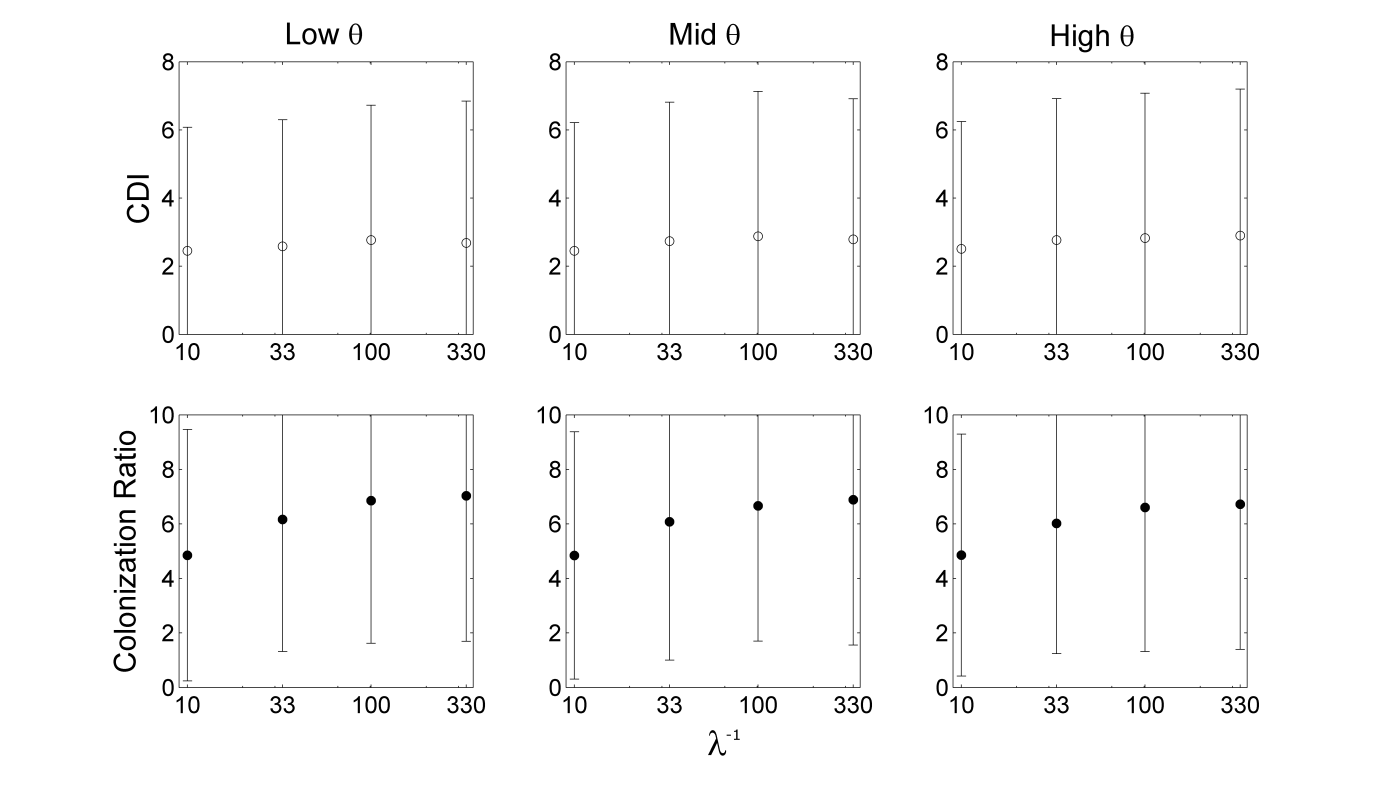


Suppl Fig 12. Probiotic gut restoration (λ^-1^) sensitivity to the rate of symptoms onset (θ).


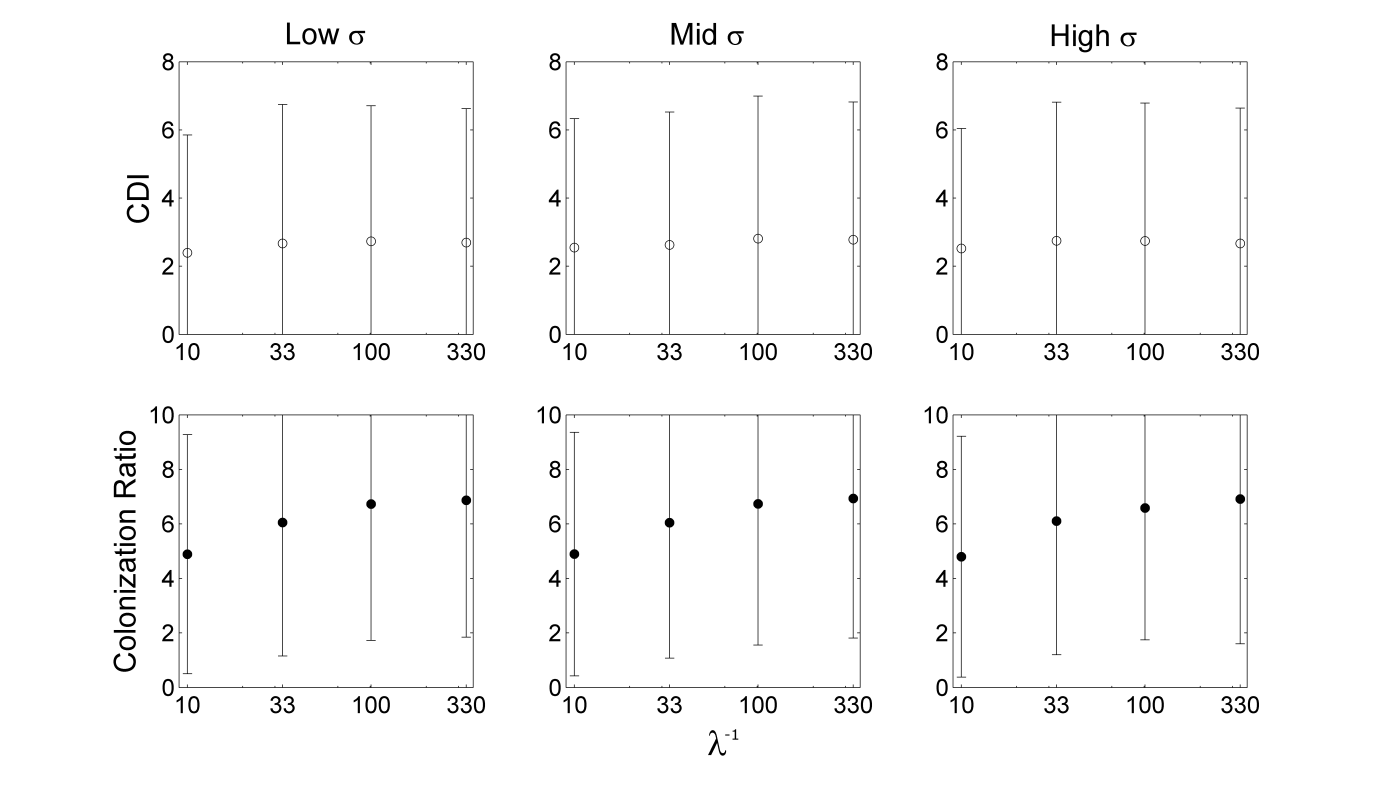


Suppl Fig 13. Probiotic gut restoration (λ^-1^) sensitivity to the CDI treatment failure rate (σ).


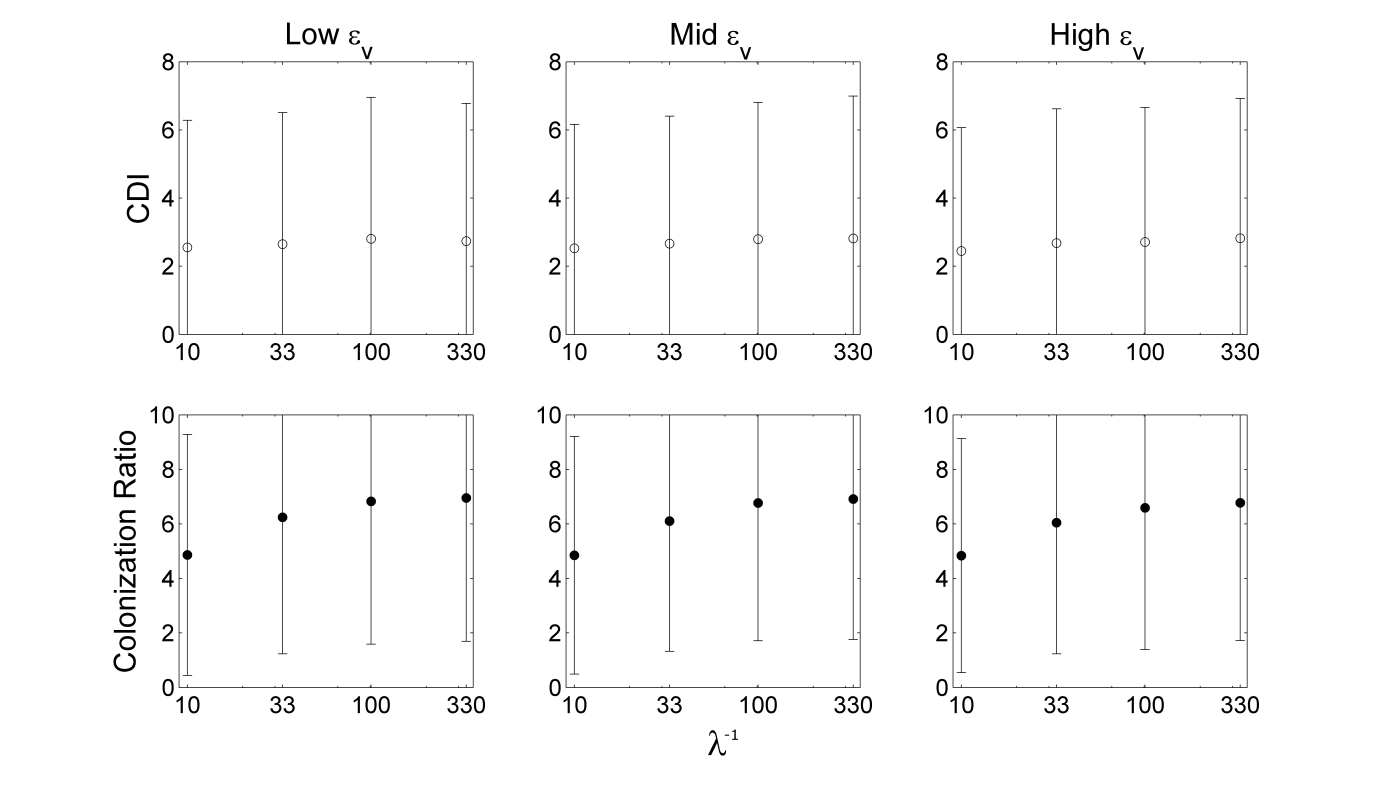


Suppl Fig 14. Probiotic gut restoration (λ^-1^) sensitivity to the proportion of vulnerable admissions (ε_v_).


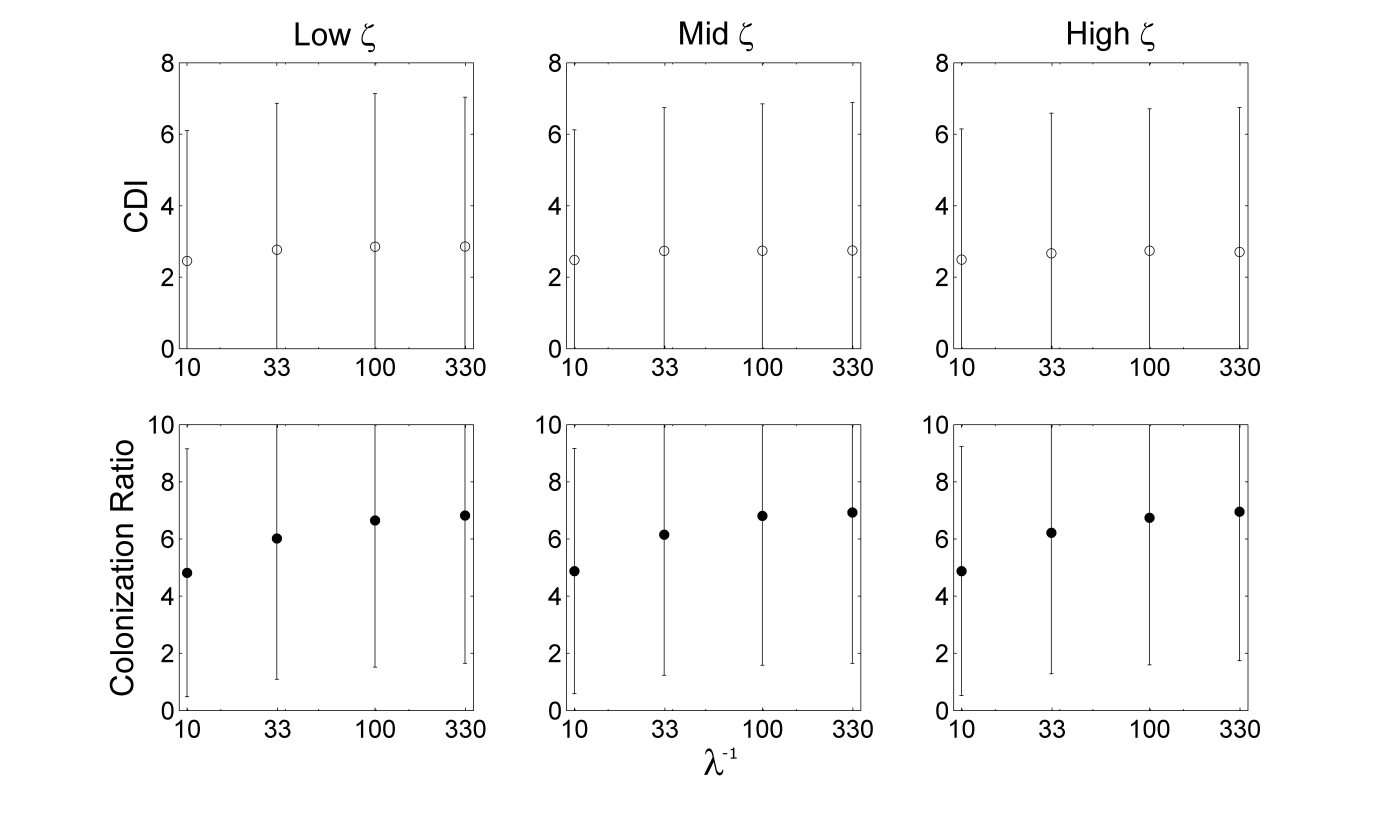


Suppl Fig 15. Probiotic gut restoration (λ^-1^) sensitivity to rate of self-resolved symptoms (ζ).


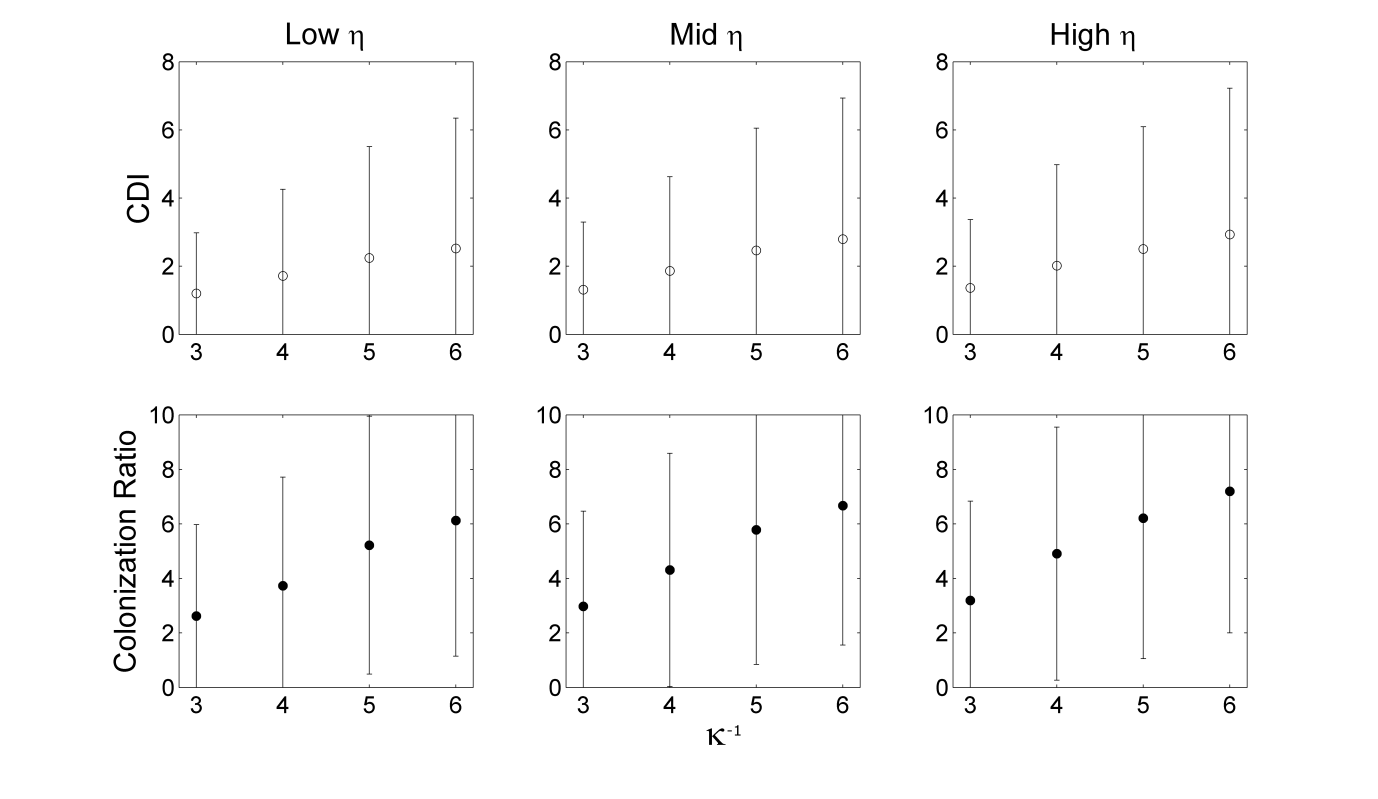


Suppl Fig 16. Reduced length of stay (κ^-1^) sensitivity to the incubation period (η).


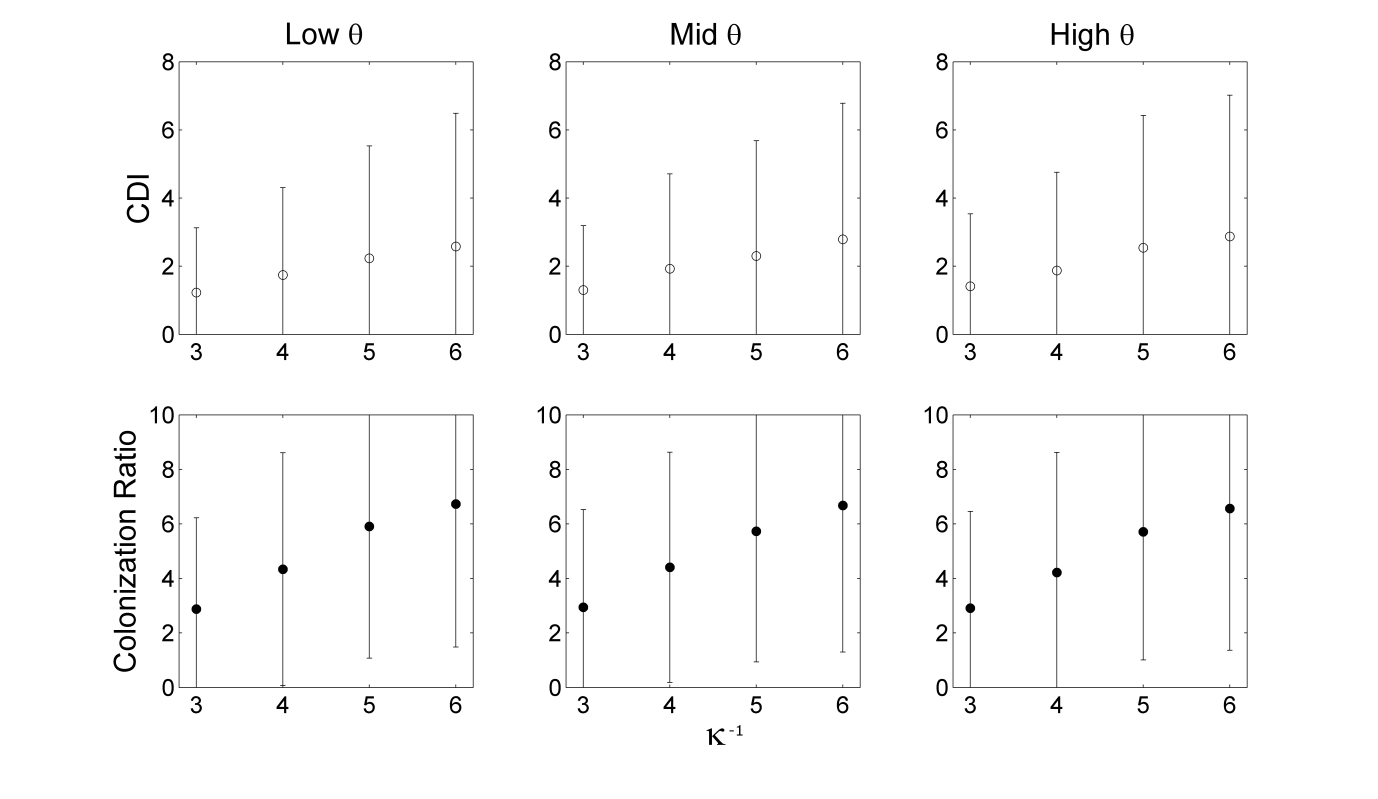


Suppl Fig 17. Reduced length of stay (κ^-1^) sensitivity to the rate of symptoms onset (θ).


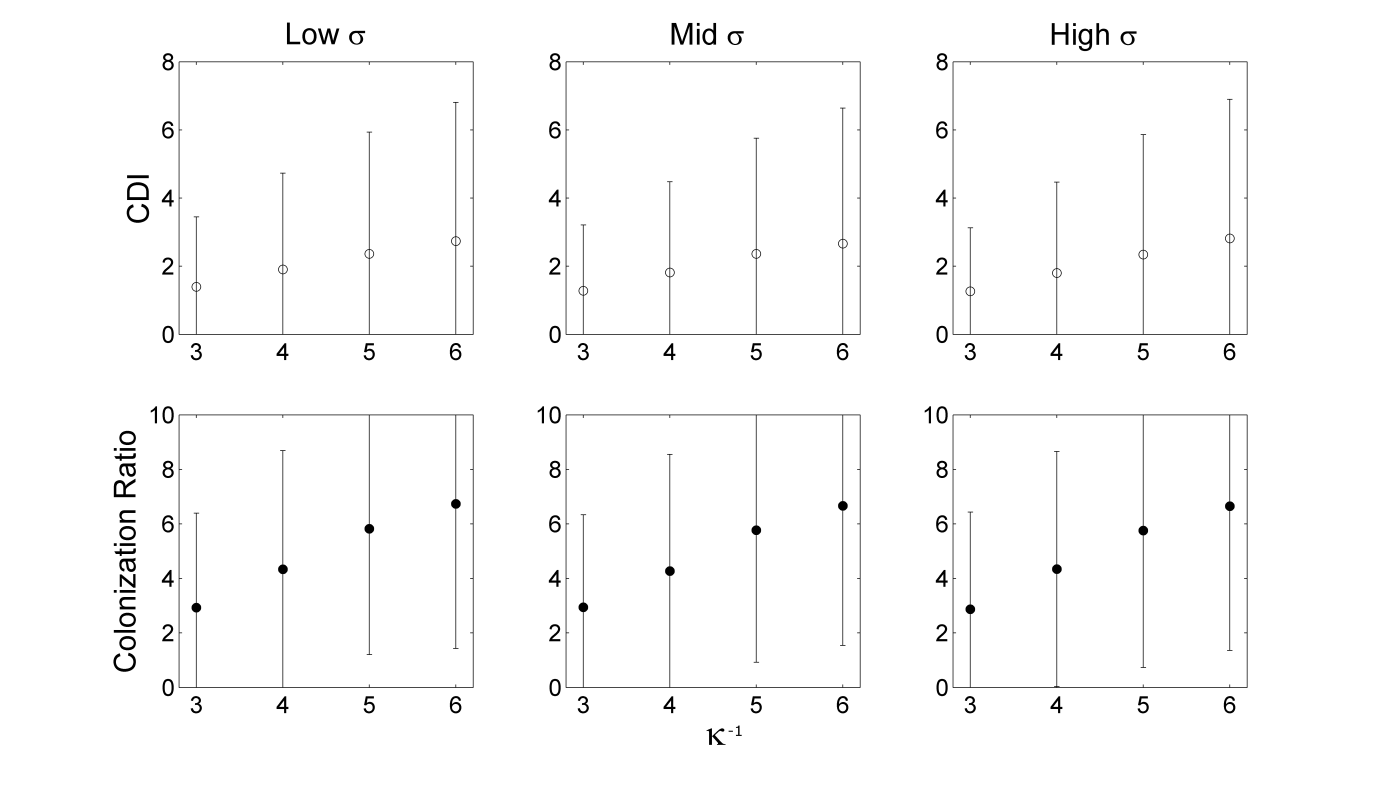


Suppl Fig 18. Reduced length of stay (κ^-1^) sensitivity to the rate of CDI treatment failure (σ).


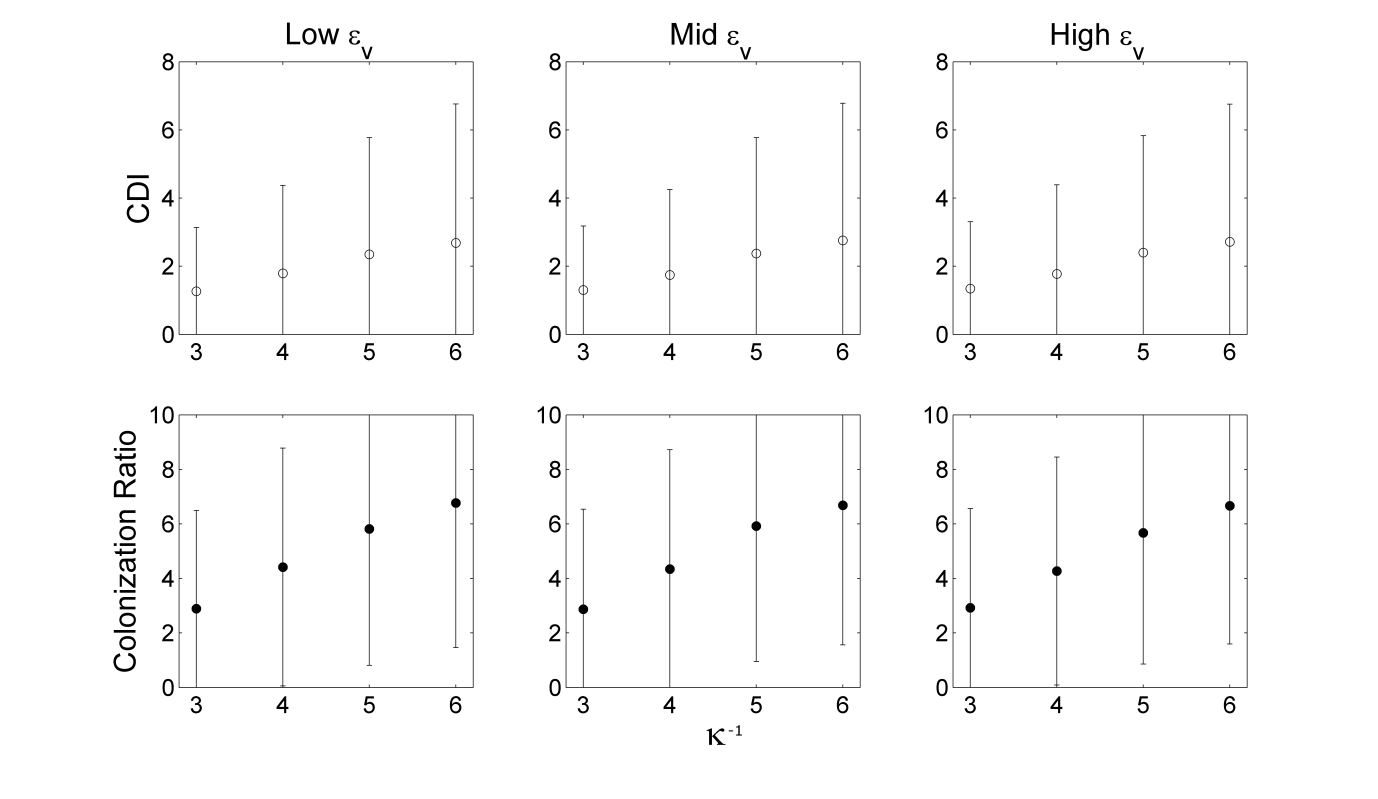


Suppl Fig 19. Reduced length of stay (κ^-1^) sensitivity to the proportion of vulnerable admissions (ε_v_).


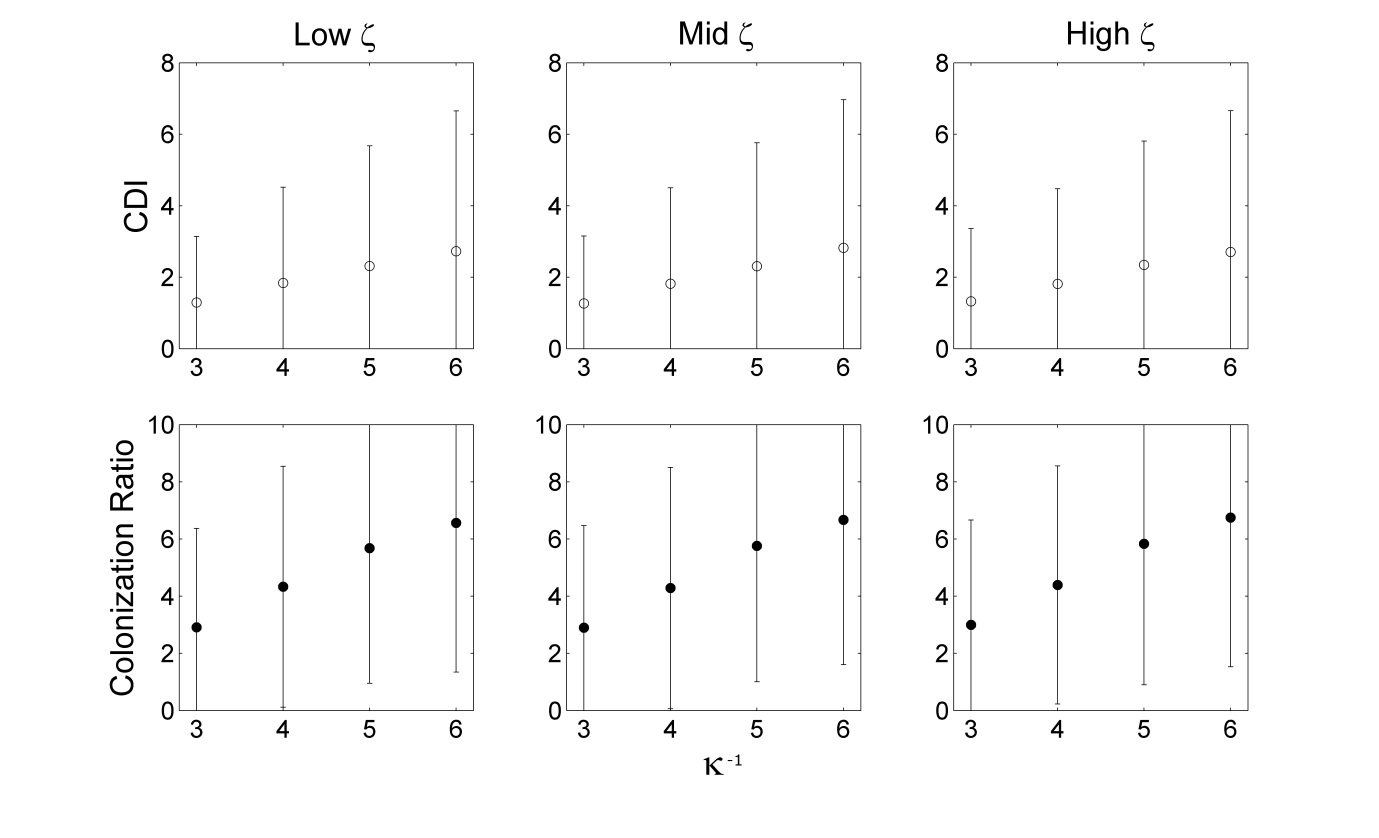
Suppl Fig 20. Reduced length of stay (κ^-1^) sensitivity to rate of self-resolved symptoms (ζ).
